# How Hyperpolarization and the Recovery of Excitability Affect Propagation through a Virtual Anode in the Heart

**DOI:** 10.1155/2011/375059

**Published:** 2011-01-13

**Authors:** Nicholas P. Charteris, Bradley J. Roth

**Affiliations:** Department of Physics, Oakland University, Rochester, MI 48309, USA

## Abstract

Researchers have suggested that the fate of a shock-induced wave front at the edge of a “virtual anode” (a region hyperpolarized by the shock) is a key factor determining success or failure during defibrillation of the heart. In this paper, we use a simple one-dimensional computer model to examine propagation speed through a hyperpolarized region. Our goal is to test the hypothesis that rapid propagation through a virtual anode can cause failure of propagation at the edge of the virtual anode. The calculations support this hypothesis and suggest that the time constant of the sodium inactivation gate is an important parameter. These results may be significant in understanding the mechanism of the upper limit of vulnerability.

## 1. Introduction

In the United States, hundreds of thousands of people die each year from sudden cardiac death, with the vast majority of those deaths caused by ventricular fibrillation. If your heart starts fibrillating, you will survive only a few minutes unless resuscitated by a strong electric shock: defibrillation. The medical device industry is a multibillion dollar business, yet defibrillators are designed empirically. Until we have a complete understanding of defibrillation, we cannot design defibrillators starting from first principles. 

Scientists study defibrillation using various tools and from a variety of perspectives [1]. Two developments in the past few decades are particularly important. The first was the discovery by Fabiato et al. [2] of the “upper limit of vulnerability” (ULV). A weak shock will not induce reentry in the heart. A stronger shock timed during the “vulnerable period” can initiate reentry, which often decays into fibrillation. Surprisingly, an even stronger shock does not produce reentry. The ULV is defined as the strongest shock that causes reentry and is often similar to the defibrillation threshold [3]. One hypothesis is that a successful defibrillation shock must not only halt preexisting fibrillation but also must not reinduce fibrillation by the mechanism for initiating reentry using a shock weaker than the ULV [4, 5]. This upper limit of vulnerability hypothesis was tested and refined in the laboratories of Ideker and Chen and has much experimental support [3–7].

The second advance was the virtual electrode hypothesis [8–11]. In 1998, Efimov et al. [12] introduced the concept of a “virtual electrode-induced phase singularity.” Shock-induced hyperpolarization deexcites cardiac tissue, creating an excitable region through which wave fronts can propagate, a “virtual anode”. After the shock, an electrotonic interaction at the border between depolarized and hyperpolarized tissue triggers a wave front, “break excitation” [13], which can only propagate in one direction into the newly created excitable region—resulting in the formation of a phase singularity and a reentrant circuit [14]. 

How does the virtual electrode-induced phase singularity hypothesis explain the ULV? Several researchers [15–18] have suggested a mechanism: a strong shock causes rapid propagation through hyperpolarized tissue, so that by the time the wave front reaches the edge of the virtual anode the surrounding tissue has not yet recovered excitability and the wave front dies. A weaker shock causes the wave front to propagate through the virtual anode more slowly, providing sufficient time for the surrounding tissue to recover. Cheng et al. [15] found that the speed of the postshock wave front depended on the magnitude of the hyperpolarization at the end of the shock and that reentry occurred only when this speed was slow. Banville et al. [16] observed similar results in their experiments, and Rodríguez and Trayanova [18] predicted analogous behavior using whole-heart numerical simulations.

These results suggest that the speed of the shock-induced wave front is crucial for determining if reentry develops. In this paper, we use a simple one-dimensional computer model to examine propagation speed through a hyperpolarized region. Our goal is to test the hypothesis that rapid propagation through a virtual anode can cause propagation to fail at the edge of the virtual anode.

## 2. Methods

We consider a one-dimensional strand of cardiac tissue governed by the cable equation

(1) $$\begin{array}{cc} C\frac{\partial V}{\partial t} & =J_{\text{stim}}-J_{\text{mem}}+\frac{g_{i}g_{e}}{\beta \left(g_{i}+g_{e}\right)}\frac{\partial^{2}V}{\partial x^{2}}, \end{array}$$

where *V* is the transmembrane potential, *J*_mem_ is the membrane current, *J*_stim_ is an applied membrane stimulus current, *C* is the membrane capacitance (0.01 F/m^2^), *g*_*i*_ and *g*_*e*_ are the intercellular and extracellular conductivities (each 0.186 S/m), and *β* is the surface to volume ratio (0.3 *μ*m^−1^). In our numerical simulation, we approximate derivatives as finite differences using an explicit method

(2) $$\begin{array}{cc} & \frac{V\left(t+\Delta t,x\right)-V\left(t,x\right)}{\Delta t}\\ & \;=\frac{1}{C}[J_{\text{stim}}-J_{\text{mem}}+\frac{g_{i}g_{e}}{\beta \left(g_{i}+g_{e}\right)}\;\\ & \;\;\;\;\;\times \frac{V\left(t,x+\Delta x\right)-2V\left(t,x\right)+V\left(t,x-\Delta x\right)}{\Delta x^{2}}]. \end{array}$$

The initial voltage is the resting potential, *V*_rest_ = −84.6 mV. The strand is 20 mm long and is sealed at the ends. The space step Δ*x* is 0.1 mm, and the time step Δ*t* is 0.005 ms.

The membrane current is calculated using the Beeler-Reuter model [19], which consists of four terms: *J*_Na_, *J*_*s*_, *J*_K1_, and *J*_*x*1_. The potassium currents *J*_*x*1_ and *J*_K1_ are both voltage dependant, and *J*_*x*1_ is also time dependant. *J*_Na_ and *J*_*s*_ are the sodium and calcium currents, where the sodium current is primarily responsible for the upstroke of the action potential. The model contains eight variables: *V*, the intracellular calcium concentration [Ca], and six ion channel gates: *m*, *h*, *j* (sodium current), *f*, *d* (calcium current), and *x*_1_ (potassium current).

In the Beeler-Reuter model, a strong hyperpolarization causes instabilities due to the exponential nature of *J*_K1_ and *J*_*x*1_. To avoid this problem, we assume that for *V* < −110 mV the currents *J*_K1_ and ${\overset{̅}{J}}_{x1}$ are linear functions of voltage [13]:

(3) $$\begin{array}{cc} & J_{\text{K}1}=-0.07656+5.329\left(V+0.110\right),\\ & {\overset{̅}{J}}_{x1}=-0.11776+6.441\left(V+0.110\right), \end{array}$$

where *J*_K1_ and ${\overset{̅}{J}}_{x1}$ are in A/m^2^, *V* is in volts, and ${\overset{̅}{J}}_{x1}$ is used in the calculation of *J*_*x*1_ by multiplying it by the gate variable *x*_1_. In addition, strong stimuli can cause [Ca] to become negative. To fix this problem, we require that [Ca] > 0 [13]. Finally, instabilities arise due to the rapid response of the ion channel gates (particularly the *m* gate) at large polarizations. The gates should stay between zero and one but sometimes deviate from this range when their time constant falls below the time step Δ*t*. To prevent this from happening, we require that all time constants be greater than or equal to Δ*t* [13].

To determine the initial conditions, we ran a sufficiently long simulation to ensure that *V*, [Ca], and all gates reached their steady-state resting values. In all other simulations, we apply a 5-ms-duration S1 stimulus to resting tissue starting at *t* = 0. The S1 stimulus *J*_stim_ = *J*_depol_ depolarizes the left-most 1 mm of tissue (0 < *x* < 1 mm). Simultaneously, the next 9 mm (1 mm < *x* < 10 mm) is hyperpolarized using a current *J*_stim_ = *J*_hyper_, with

(4) $$\begin{array}{c} J_{\text{hyper}}=-\frac{J_{\text{depol}}}{\alpha }, \end{array}$$

where *α* = 9. This hyperpolarized region simulates the “virtual anode” observed during unipolar cardiac stimulation [20] and found in Efimov et al.'s experiments [12]. The region 10 mm < *x* < 20 mm is not stimulated (*J*_stim_ = 0). The stimulus threshold for resting tissue is *J*_depol_ = 0.0633 A/m^2^. For all simulations besides those to find the resting threshold, we fix S1 as twice the threshold, *J*_depol_ = 0.127 A/m^2^.

The first stimulus creates an action potential that propagates down the strand. We apply a second 5-ms stimulus, S2, beginning at time *t*_2_ near the end of the S1 action potential's refractory period. Again, the region 0 < *x* < 1 mm is depolarized, and the region 1 mm < *x* < 10 mm is hyperpolarized, with the depolarization stimulus current nine times as strong as the hyperpolarization stimulus current. In simulations using a higher pacing rate, ten S1 stimuli are applied every 400 ms, followed by S2. In one simulation, S1 is uniform (*J*_stim_ = *J*_depol_ over the entire strand 0 < *x* < 20 mm), but S2 is as described earlier.

The propagation speed *u* is determined by finding the time *t*_max_ when *dV*/*dt* is maximum (during the upstroke) for each point *x* and then calculating

(5) $$\begin{array}{c} u\left(x\right)=\frac{2\Delta x}{t_{\max\;}\left(x+\Delta x\right)-t_{\max\;}\left(x-\Delta x\right)}. \end{array}$$

In finding the times with maximum *dV*/*dt*, we ignore the first 5 ms after the end of the S2 stimulus, and do not consider times when the potential is below −60 mV, because at these times a large *dV*/*dt* is usually caused by the recovery from hyperpolarization and not by a propagating action potential.

## 3. Results

**Figure 1:**
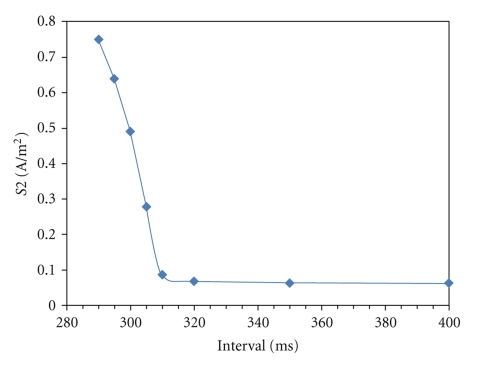
Strength-interval curve: the minimum necessary S2 stimulus strength to excite a propagating action potential for various S1-S2 intervals.

Figure 1 shows the strength-interval curve for the S2 stimulus. After about 320 ms, the curve is nearly flat and approaches the threshold for resting tissue. For earlier times, the threshold stimulus is higher, reflecting refractoriness from the S1 action potential. 

**Figure 2:**
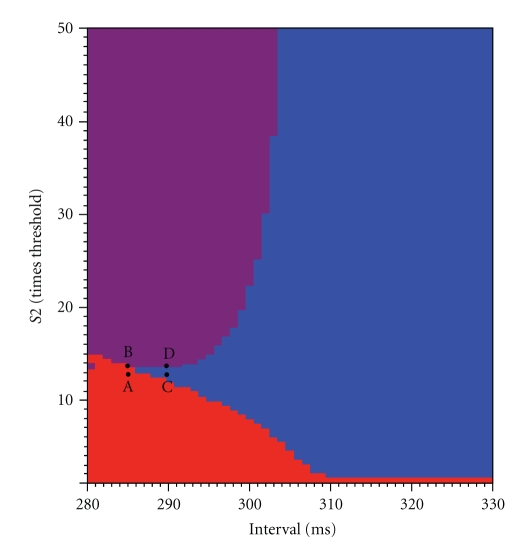
The behavior as a function of the S2 stimulus strength and the S1-S2 interval. Blue indicates that the S2 action potential propagated across the entire 20 mm strand, purple indicates that the S2 action potential propagated about halfway (to the edge of the hyperpolarized region) and then died, and red indicates that the S2 stimulus failed to excite an action potential. The points A, B, C, and D correspond to the four simulations shown in more detail in Figure 3.

The fate of the S2 action potential is shown along with the strength-interval curve in Figure 2, for much stronger stimuli. The vertical axis indicates the stimulus strength divided by the threshold strength for resting tissue, and the plot shows S2 strengths up to 50 times threshold. Red indicates that the S2 stimulus did not fire an action potential. Blue indicates that an action potential propagated across the entire strand (to *x* = 20 mm). Of particular interest is the region corresponding to strong stimuli and short intervals (purple), when the S2 action potential propagated to the edge of the virtual anode (*x* = 10 mm) and then died. If we take our criterion for a “successful” response to the S2 stimulus as propagation all the way to the right edge of the strand, then for many intervals there is a range of stimulus strengths that are successful, and stimuli outside this range (either higher or lower) fail. For instance, at an interval of 300 ms, the S2 stimulus is successful over a range from about 8 to 20 times threshold. 

**Figure 3:**
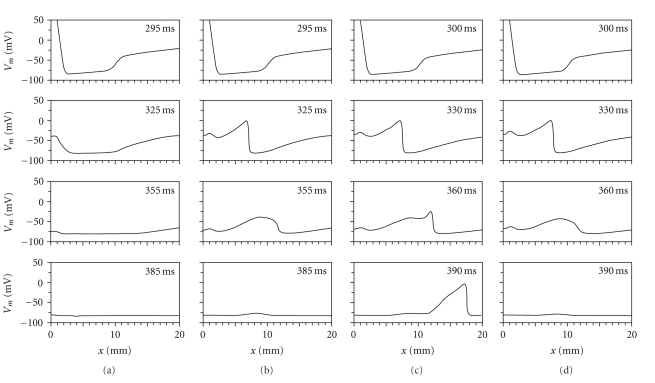
Voltage *V* as a function of position *x*, at four times. (a) For an S2 of 13 times threshold at *t*_2_ = 285 ms, the stimulus does not excite an action potential. (b) For an S2 of 14 times threshold at *t*_2_ = 285 ms, an S2 action potential propagates along the strand until about *x* = 10 mm, after which it dies. (c) For an S2 of 13 times threshold at *t*_2_ = 290 ms, an S2 action potential propagates along the entire strand. (d) For an S2 of 14 times threshold at *t*_2_ = 290 ms, an S2 action potential propagates along the strand until *x* = 10 mm and then dies.

To understand better the fate of the S2 action potential, we plot *V* versus *x* at several times in Figure 3, corresponding to the four points A, B, C, and D in Figure 2. In Figure 3(a), the S2 stimulus is applied at *t*_2_ = 285 ms and has a strength of 13 times threshold. The upper curve is drawn at *t* = 295 ms, soon after the S2 stimulus ends. The large depolarization on the left is caused directly by the stimulus, as is the weaker hyperpolarization in the range 1 mm < *x* < 10 mm. At later times, the depolarization on the left dies away without exciting an action potential (the tissue was refractory), a behavior corresponding to the red region in Figure 2. In Figure 3(b), the stimulus is slightly stronger (14 times threshold), and an action potential is excited (see *t* = 325 ms), but it fails to propagate much beyond *x* = 10 mm, an example of the purple region in Figure 2. In Figure 3(c), the S2 stimulus (13 times threshold) is applied slightly later (*t*_2_ = 290 ms), and the action potential propagates successfully across the entire strand, corresponding to the blue region in Figure 2. A small increase in the stimulus strength (14 times threshold) at the same time (*t*_2_ = 290 ms), shown in Figure 3(d), results in a failure to propagate at the edge of the virtual anode.

Figure 3 raises an interesting question: why did the S2 action potential propagate successfully to the end of the strand in some cases but die at the edge of the virtual anode in others, a behavior corresponding to the boundary dividing the blue and purple regions in Figure 2? A change in the S1 refractoriness plays a role, because the boundary depends on the interval. However, even at a fixed interval increasing the S2 stimulus strength can cause propagation to fail. In order to explore the mechanism underlying this behavior, we examine the propagation speed as a function of position.

**Figure 4:**
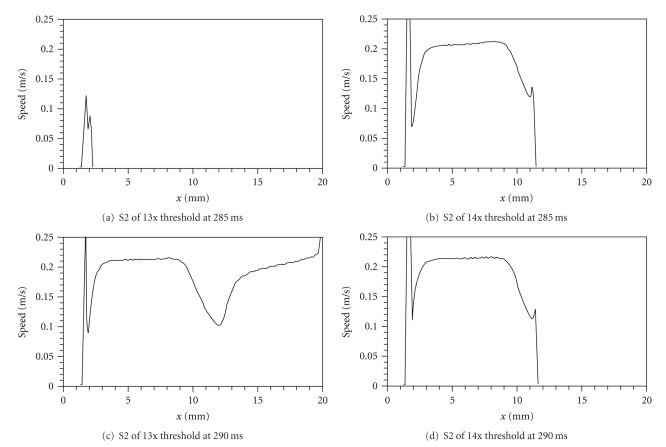
Calculated action potential speed as a function of position, for the simulations shown in Figure 3.

In Figure 4(a), the subthreshold S2 stimulus fails to excite an action potential, so the speed is zero except near the left edge, where diffusion of the depolarization caused by the stimulus masquerades as propagation. In each of the other three cases (Figures 4(b)–4(d)), the speed in the hyperpolarized region is about 0.21 m/s (except for an initial transient associated with the stimulus). The wave front slows near the edge of the virtual anode (*x* = 10 mm) and then either dies there (Figures 4(b) and 4(d)) or propagates successfully through the slow region and afterwards recovers its speed (Figure 4(c)). However, there is not an obvious difference of the propagation speed within the virtual anode between the two simulations using an S2 stimulus at *t*_2_ = 290 ms (Figures 4(c) and 4(d)).

**Figure 5:**
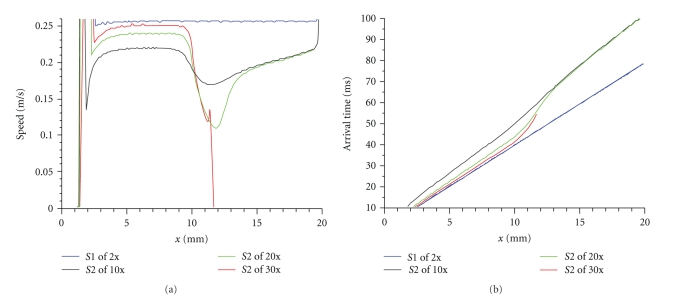
(a) Propagation speed and (b) arrival time, for an S2 stimulus applied at *t*_2_ = 300 ms with an S2 strength of 10 (gray), 20 (green), and 30 (red) times threshold. The speed and arrival time of the S1 action potential (blue) are shown for comparison.

Because the stimuli used in Figures 3 and 4 are so similar, it is difficult to detect any difference in the maximum speed through the hyperpolarized region (all are about 0.21 to 0.22 m/s). To clarify the relationship between stimulus strength and propagation speed, we compare speeds for three very different S2 stimulus strengths (Figure 5(a)). All three S2 action potentials have speeds that are slower than the speed of the S1 action potential, which traveled about 0.25 to 0.26 m/s. In fact, even for very strong stimuli (50–100 times resting threshold), the S2 propagation speed through the hyperpolarized tissue never rises above 0.26 m/s. Therefore, it is incorrect to say that the hyperpolarization hastens propagation through the virtual anode compared to the speed of the S1 action potential. However, the degree of slowing in the virtual anode caused by S1 refractoriness is reduced as the S2 stimulus strength increases.

Another interesting feature of Figure 5(a) is the difference between the speed of the S2 wave front within the virtual anode and at its edge. At 10 times threshold propagation is significantly slowed in the virtual anode, but the additional slowing at the edge of the virtual anode is not great. On the other hand, at 20 times threshold propagation in the virtual anode is somewhat faster than for the weaker S2 stimulus, but the slowing at the edge of the virtual anode is quite dramatic. For 30 times threshold the speed within the virtual anode is further increased, so that it is only slightly slower than the S1 action potential, but the slowing at the edge of the virtual anode is so marked that propagation fails. Thus, increasing the S2 stimulus strength causes two competing effects: it increases speed within the virtual anode but decreases it at the edge. 

To sort out which of these effects is dominant, Figure 5(b) shows the arrival time of the action potential as a function of distance. In this plot, a slower speed corresponds to a steeper slope. Clearly the increase in speed through the virtual anode is the more important effect, as it results in a shorter arrival time for strong stimuli. Another factor may be the location where the action potential originates. For stronger S2 shocks the action potential starts at larger values of *x*, essentially getting a “head start” in its race across the virtual anode (this is sometimes called the “virtual cathode” effect [21]). The arrival time of the S2 wave front at the edge of the virtual anode is the crucial factor and is determined by both the speed and origin of the action potential. When the arrival time is delayed enough that the surrounding tissue has time to recover excitability, propagation success is more likely.

**Figure 6:**
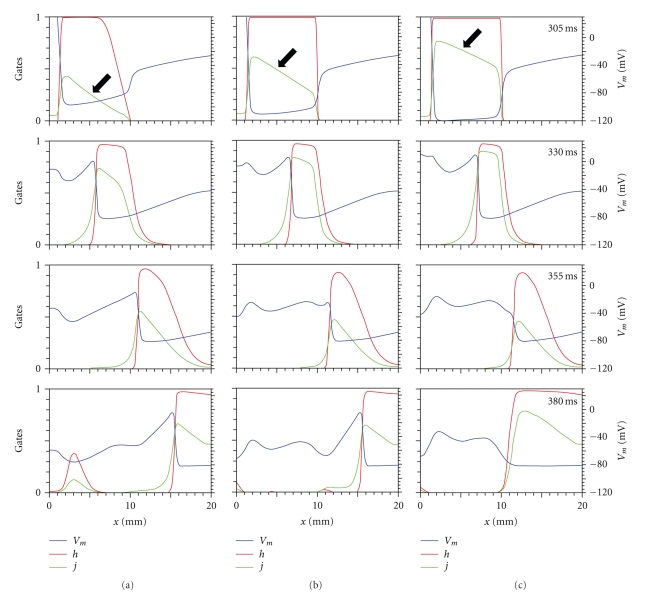
The voltage *V* (blue) and the sodium channel inactivation gates *h* (red) and *j* (green) as functions of position *x*, for the times indicated at the top of each frame (in ms). The S2 stimulus is applied at *t*_2_ = 300 ms and has a strength of (a) 10, (b) 20, and (c) 30 times threshold.

If recovery of excitability is indeed the key for propagation success, we should see differences in the inactivation of the sodium channel (the main influence on excitability) as we vary the S2 stimulus strength. In the Beeler-Reuter model [19], the sodium channel has two inactivation gates—*h* and *j*—having similar properties except that *j* has a slower time constant than *h*.  Figure 6 shows *V*, *h*, and *j* as functions of position for various times. For the three S2 stimulus strengths we examine, the hyperpolarization of the virtual anode is sufficient to open *h* completely (*x* < 10 mm, *t* = 305 ms, just at the end of the S2 shock), and it remains open until the S2 action potential passes by (*t* = 330 ms). In the region outside the virtual anode (10 mm < *x* < 20 mm) *h* is closed during and immediately after the shock (*t* = 305, 330 ms); the tissue is refractory from the S1 action potential, and the S2 stimulus has little effect. Only at about *t* = 355 ms does this region begin to recover excitability. The dramatic difference in S2 stimulus strength of the three simulations in Figure 6 results in only small differences in the *h* gate in the virtual anode (*t* = 305 ms). However, because of its longer time constant, the hyperpolarization in the virtual anode is not sufficient to drive the slow sodium inactivation gate, *j*, completely open. Instead, its value in the virtual anode depends strongly on the S2 stimulus. Thus, the excitability of the tissue in the virtual anode is greater for stronger S2 stimuli (there is a larger value of *j* at *t* = 305 ms, *x* < 10 mm). To more clearly see this, compare the *j* trace (green curve) in the top panels (*t* = 305 ms) for each of the three columns (for S2 stimuli of 10, 20, and 30 times threshold) of Figure 6. The key point is that the value of *j* in the virtual anode (e.g., look at *x* = 5 mm) increases as the S2 stimulus increases, from *j* = 0.3 for 10 times threshold, to *j* = 0.5 for 20 times threshold, to *j* = 0.7 for 30 times threshold (see arrows in Figure 6). The propagation speed is therefore faster for strong stimuli; at 330 ms the action potential for the 10x stimulus has reached about *x* = 5.8 mm, while for the 30x stimulus it has already reached *x* = 7.2 mm. At *t* = 355 ms, when the S2 wave front initiated by the weak shocks (Figures 6(a) and 6(b)) reaches the edge of the virtual anode, the tissue adjacent to the virtual anode (about *x* = 11 mm) has recovered excitability sufficiently to support propagation. For a strong shock (Figure 6(c)) the wave front arrived before 355 ms, failed at the edge of the virtual anode, and in the *t* = 355 ms frame the wave front has already begun to decay. The *t* = 380 ms plots show successful propagation past the edge of the virtual anode in Figures 6(a) and 6(b) and failure in Figure 6(c). 

To determine if the S1 pacing rate has any influence on the results, we repeat our simulations using ten S1 pacing stimuli each separated by 400 ms. The results are qualitatively the same, although the strength-interval behavior of Figure 2 is shifted toward shorter intervals by about 40 ms. This observation is consistent with the results of Bennett and Roth [22], who found that the strength-interval curve for a similar situation was unchanged except for a shift to shorter intervals when the S1 pacing rate was increased. We also perform simulations in which S1 is delivered along the entire strand simultaneously (with S2 unchanged from that described earlier). Again, the qualitative results are not changed by the elimination of the S1 refractory gradient, but quantitatively the strength-interval curve shifts to shorter intervals, reflecting the propagation time across the virtual anode (about 40 ms). This is consistent with previous studies of virtual electrode-induced reentry, in which the location and polarity of the S2 reentrant circuit was nearly independent of the S1 refractory gradient [23–25].

## 4. Discussion

Our simulations support the hypothesis that the speed of propagation through the virtual anode is a key factor in propagation success. If the speed is slow (because the S2 shock did not completely restore tissue excitability), the surrounding tissue that is not affected by the shock has more time to recover excitability, making propagation from the virtual anode into the surrounding tissue possible. A stronger S2 stimulus applied to refractory tissue leads to a greater hyperpolarization, which results in a greater recovery of excitability, implying a faster speed, thereby increasing the probability of propagation failure at the virtual anode edge. This behavior is consistent with a previous explanation for the mechanism of the “no-response” phenomenon in cardiac tissue [14, 26], with previous suggestions for the mechanism of the ULV [15–18], and with calculations suggesting that “the fate of the shock-induced break wave front when it reached the edge of the virtual anode was found to be the key to understanding the ULV” [27].

The variations in the sodium inactivation gates *h* and *j* influence excitability, explain the differences in speed in Figure 4, and thereby determine propagation success or failure. In normal resting tissue, both *h* and *j* are nearly one (this may not be true for tissue in which the resting potential has been elevated by, for instance, high extracellular potassium [28]). Thus, the excitability of the hyperpolarized tissue following an S2 shock cannot be greater than the excitability of resting tissue: the excitability is the greatest when both *h* and *j* are one and cannot get any greater. However, when the S2 shock is applied to refractory or incompletely recovered tissue—such as often present in the excitable gap of a reentrant circuit [29, 30]—the strength of the hyperpolarization influences how well the stimulus can force the tissue to recover from refractoriness. The main factor appears to be the *j* gate, because its slower time constant does not allow it to recover excitability quickly. Other gates—such as the inactivation gate for the calcium current, *f*—do not change significantly in response to a 5-ms-long hyperpolarization because of their slow time constant and therefore play a minor role in determining the response of the tissue to hyperpolarization. The state of the tissue before the S2 shock (e.g., during rapid pacing) also plays a role in determining the recovery of excitability.

The calculations presented here have several limitations. (1) The model is based on a 1-dimensional approximation of cardiac tissue. We cannot look at reentry, which is inherently a two- or three-dimensional event, in these simulations, so we cannot directly calculate the ULV. Also, other factors that influence propagation speed, such as wave front curvature, are absent in our calculations. Nevertheless, by using a simple one-dimensional model, we are able to isolate and focus on the mechanism of recovery from refractoriness without additional confounding factors such as wave front curvature. Our model predicts ULV-like behavior without wave front curvature, suggesting that curvature is not an essential element of the ULV mechanism. (2) The preshock state is much simpler than fibrillation, which we cannot model using a one-dimensional cable. However, the behavior in our simulations is qualitatively similar when using rapid S1 pacing rates, and when S1 is uniform throughout the tissue, suggesting that our conclusions are not sensitive to the preshock state of the tissue. (3) The effect of the S2 stimulus is represented by an artificial distribution of membrane current (strongly depolarizing for 0 < *x* < 1 mm and weakly hyperpolarizing for 1 mm < *x* < 10 mm, with no effect for 10 mm < *x* < 20 mm). While this distribution is reminiscent of the shock distribution observed by Efimov et al. [12], it is certainly not equivalent to their observation. Our goal is to test if an extremely simple, idealized model for a shock can explain the mechanism of the ULV. While our results are suggestive, additional simulations using a more realistic model are necessary before any final conclusions can be drawn. Factors such as the size of the virtual anode and the sharpness of the gradient between depolarized, hyperpolarized, and unaffected regions may be important. (4) The Beeler-Reuter model is used to represent the ion channel kinetics, rather than more modern models (e.g., [31]). In particular, the Beeler-Reuter representation of the potassium and calcium currents has been improved in more recent models. Additional studies need to be performed to see if these results generalize to other membrane models, particularly ones with different sodium channel properties. Nevertheless, our results suggest that the time constant of the sodium channel inactivation gate may be important for determining how hyperpolarization causes the wave front to propagate through the virtual anode. Factors such as drugs that influence this time constant may play a key role in determining the upper limit of vulnerability, and thus the defibrillation threshold. Also, our results suggest that the ULV may be sensitive to the S2 shock duration, because increasing the duration would lengthen the time available for the shock to remove sodium channel inactivation and thereby increase excitability in the virtual anode, implying that the wave front is more likely to fail at the edge of the virtual anode, corresponding to defibrillation success.
